# Supplementary material for: Neural categorization of visual words of alphabetic and non-alphabetic languages
Source: eLife. 2026 Jul 20;15:RP110320. doi: 10.7554/eLife.110320 (PMC13384499; doi:10.7554/eLife.110320)
Supplement: Peer review history

# Editor report: eLife Assessment

**DOI:** 10.7554/eLife.110320.3.sa0

**Authors:** Nai Ding

**Affiliations:** Zhejiang University China

**Keywords:** Important, Solid

This **important** study investigates how the brain categorizes written words from different writing systems (e.g., alphabetic vs. non-alphabetic). The evidence supporting the authors' claims is **solid** and sheds light on the neural basis of language's social‑categorization function.

# Referee report: Reviewer #1 (Public review)

**DOI:** 10.7554/eLife.110320.3.sa1

Summary:

This study demonstrates, through a series of EEG and MEG experiments, that the human brain automatically categorizes words from alphabetic and non-alphabetic languages, and it unpacks the neural mechanisms of this process from multiple angles. The work examines not only univariate repetition-suppression (RS) effects, but also how repeating or alternating languages influences the representational similarity of words within and across language categories.

Strengths:

The univariate RS effects across multiple experiments lend support to some of the main conclusions.

Comments on revised version.

The authors have made appropriate revisions and supplements in response to the issues I raised, which has largely resolved my concerns.

# Referee report: Reviewer #2 (Public review)

**DOI:** 10.7554/eLife.110320.3.sa2

Summary:

This study investigates how the human brain categorizes visual words from distinct writing systems (alphabetic vs. non-alphabetic). Using a repetition suppression paradigm combined with electroencephalography and magnetoencephalography, the authors conducted nine experiments with independent participants to identify the neural network underlying language-based categorization, characterize its temporal dynamics, and test whether this process operates independently of linguistic properties such as semantic meaning and pronunciation.

Strengths:

The study employs a well-validated design with clear control conditions and systematically manipulates key variables including writing system, language familiarity, and native language background. The use of nine experiments with independent participant samples strengthens the reliability and replicability of the results. The work combines EEG and MEG, cross-validating findings across imaging modalities to support the reported neural effects. A combination of univariate, multivariate, and connectivity analyses is used to characterize neural responses and network interactions. Results are consistent across multiple language groups and for both familiar and unfamiliar languages, supporting the generalizability of the identified neural mechanism beyond specific languages or prior experience.

Comments on revised version.

Earlier versions of the manuscript framed these findings as more directly reflecting the social-categorization function of language. In the revised manuscript, the authors now more carefully distinguish language-based word categorization from broader claims regarding social categorization and explicitly acknowledge that the current experiments do not directly test social evaluation or intergroup processes. These revisions improve the conceptual precision of the work and address my major concern from the previous review.

The additional methodological clarifications and supplementary analyses also strengthen the manuscript. Overall, I believe the revised version provides solid evidence for rapid language-based categorization of visual words across different writing systems.

# Author comment: Author response

**DOI:** 10.7554/eLife.110320.3.sa3

**Authors:** Guo Zheng, Shihui Han

**Affiliations:** Peking University Beijing China; Peking University Beijing China

The following is the authors’ response to the original reviews.

**eLife Assessment**

This important study investigates how the brain categorizes written words from different writing systems (e.g., alphabetic vs. non-alphabetic), shedding potential light on the neural basis of language's social‑categorization function. Overall, the evidence supporting the authors' claims is solid, though some analyses and key interpretations would benefit from fuller justification.

Thank you for handling our manuscript! We’ve modified the manuscript according to the reviewers’ comments and suggestions.

**Public Reviews:**

**Reviewer #1 (Public review):**

Summary:

This study demonstrates, through a series of EEG and MEG experiments, that the human brain automatically categorizes words from alphabetic and non-alphabetic languages, and it unpacks the neural mechanisms of this process from multiple angles. The work examines not only univariate repetition-suppression (RS) effects, but also how repeating or alternating languages influences the representational similarity of words within and across language categories.

Strengths:

The univariate RS effects across multiple experiments lend support to some of the main conclusions

Weaknesses:

I have reservations about the logic underlying the multivariate analyses, and I believe the implications of the control experiments merit fuller discussion.

(1) Question 1: Logic of the multivariate analyses

The original text states:

"The processing of intra-language similarity was quantified as correlation distances between neural responses to two words of the same language, which occurred more frequently and would be inhibited in the Rep-Cond (vs. Alt-Cond) due to habituation (Fig. 1c)...".

I argue that this passage conflates two levels. Building a representational dissimilarity matrix (RDM) is a data-analysis step; it cannot be equated with a cognitive computation. Hence, there is no sense in which this computation occurs "more frequently" in one condition. RDM construction rests on the pairwise similarity of activity patterns, so even if a task engaged no cognitive computation of representational similarity, we could still compute an RDM. Conversely, if a task factor alters the RDM, we must explain how that factor changes the underlying neural patterns, not claim that it triggers specific cognitive processing. Therefore, I neither understand what "more frequent processing" the authors refer to, nor accept their account of the multivariate results.

The multivariate result pattern, briefly, is that distances between words, both within and across languages, are larger under the repetition condition. One plausible interpretation is that a word representation comprises two parts: language-type (alphabetic vs. non-alphabetic) and fine-grained identity features (visual shape, orthography, semantics, phonology, etc.). Repetition of language type may, via RS, reduce the weight of the first component, thereby increasing the relative contribution of fine-grained features and amplifying inter-word differences. This could explain the multivariate findings.

Thank you for these insightful comments regarding the logic of the multivariate analyses. In the revision, we’ve elaborated the rationale underlying our experimental design. Specifically, we’ve explained why the processing of intra-language similarity is expected to occur more frequently in the repetition condition (Rep-Cond) than in the alternation condition (Alt-Cond) whereas the reverse is true for the processing of inter-language difference. Importantly, we’ve clarified that the processing of intra-language similarity was assessed rather than defined by conducting the multivariate analyses. The multivariate analyses were conducted to assess correlation distances between neural responses to pairs of words, either within the same language or across different languages. We explained what smaller intra-language correlation distances and larger inter-language correlation distances mean for language-base categorization of words (see Page 7-8).

We appreciate the alternative account of the observed neural repetition suppression (RS) effects in terms of language-type versus fine-grained identity (visual shape, orthography, semantics, phonology, etc.) feature processing. We included a paragraph in the revised Discussion to discuss how possible the early neural RS effect can be attributed to the processing of the fine-grained identity features of visual words. This discussion allowed us to clarify that the early neural RS effects related to visual words of familiar and unfamiliar languages highlight the early spontaneous language-based categorization as a unique process of visual words of alphabetic and non-alphabetic languages. However, our results do not exclude the possibility that the processing of the linguistic properties of visual words may contribute to the long-latency RS effect (see Page 37-38).

Page 7-8

“The processing of intra-language similarity occurs when two words of the same language are perceived repeatedly with short interstimulus intervals. Because words of the same language were repeatedly presented in the Rep-Cond and words of two different languages were displayed in the Alt-Cond, the processing of intra-language similarity occurred more frequently and would be inhibited in the Rep-Cond (vs. Alt-Cond) due to habituation (Fig. 1c). By contrast, the processing of inter-language difference takes place when two words of different languages are perceived with short interstimulus intervals. Since words of different languages appeared more frequently in the Alt-Cond (vs. Rep-Cond), we would expect RS of the processing of inter-language difference in the Alt-Cond (vs. Rep-Cond). The neural processing of intra-language similarity was quantified as correlation distances between neural responses to two words of the same language whereas the neural processing of inter-language difference was assessed as correlation distances between neural responses to two words of two different languages. The correlation distances from the multivariate analyses were further employed to assess how words of one language are clustered and how far words of two languages are separated in a two-dimensional (2D) space during language-based word categorization. Enhanced language-based word categorization is associated with smaller intra-language correlation distances, which reflect more densely clustered words of the same language, and larger inter-language correlation distances, which manifest further separated words of two different languages.”

Page 37-38

“How possible are the early neural RS effects within 200 ms after word onset observed in our study related to the processing of low-level perceptual features or high-level linguistic (e.g., orthography, semantics, phonology) properties of visual words? Our analyses of the ERPs to scrambled Chinese and English words in Experiment 2 did not show significant RS effect. Because only low-level visual features were preserved in the scrambled words, the ERP results provided no evidence that the early RS effects on the neural response to words can be attributed to habituation of perception of the low-level perceptual features. Furthermore, we found that the RS effects on the neural response to radicals and letters in Experiment 3 took place in a delayed time window and exhibited different scalp distributions (i.e., over the central region for radicals and occipital regions for letters) compared with the neural RS effects related to words. Thus the early RS effects on the neural response to words cannot be interpreted as habituation of perception of the middle-level units of Chinese and English words (i.e., radicals and letters) either. In addition, the early neural RS effects were similarly observed for both familiar (i.e., Chinese and English) and unfamiliar (i.e., Korean and Italian) languages and occurred earlier than the time window in which the processing of the linguistic properties of visual words takes place (Marinkovic et al., 2003; Hodgson et al., 2021; Zhu et al., 2022). Therefore, the early neural RS effects identified in our work were unlikely to be associated with the processing of the linguistic (e.g., orthography, semantics, phonology) properties of visual words since these properties of unfamiliar languages were unknown to the participants. Taken together, our findings of the early neural RS effects highlight an early word-level representation of alphabetic vs. non-alphabetic languages which distinguishes words from letters/radicals but is similar for familiar or unfamiliar languages. Our results, however, do not exclude the possibility that the processing of the linguistic properties of visual words may contribute to the long-latency RS effect around 300 ms after word onset. Further processing of the linguistic properties of visual words of familiar languages may follow the early language-based categorization of visual words, though this should be tested in future research.”

(2) Question 2:

For unlearned languages, people cannot distinguish lexical from sub-lexical levels. What, then, determines (i) the RS-effect difference between letters and radicals in familiar languages and words in unlearned ones, and (ii) the similarity of repetition effects between words in unlearned and familiar languages? An explicit account is needed.

Thank you for this suggestion. In the revised manuscript, we’ve included a dedicated paragraph addressing these two issues. Specifically, we’ve provided a more precise account of the differences in repetition suppression (RS) effects between words and letters/radicals in familiar languages, as well as the similar RS effects observed for unlearned and familiar languages. We believe that our findings of the early neural RS effects highlight an early word-level representation of alphabetic vs. non-alphabetic languages which distinguishes words from letters/radicals but is similar for familiar or unfamiliar languages (see Page 37-38).

Page 37-38

“How possible are the early neural RS effects within 200 ms after word onset observed in our study related to the processing of low-level perceptual features or high-level linguistic (e.g., orthography, semantics, phonology) properties of visual words? Our analyses of the ERPs to scrambled Chinese and English words in Experiment 2 did not show significant RS effect. Because only low-level visual features were preserved in the scrambled words, the ERP results provided no evidence that the early RS effects on the neural response to words can be attributed to habituation of perception of the low-level perceptual features. Furthermore, we found that the RS effects on the neural response to radicals and letters in Experiment 3 took place in a delayed time window and exhibited different scalp distributions (i.e., over the central region for radicals and occipital regions for letters) compared with the neural RS effects related to words. Thus the early RS effects on the neural response to words cannot be interpreted as habituation of perception of the middle-level units of Chinese and English words (i.e., radicals and letters) either. In addition, the early neural RS effects were similarly observed for both familiar (i.e., Chinese and English) and unfamiliar (i.e., Korean and Italian) languages and occurred earlier than the time window in which the processing of the linguistic properties of visual words takes place (Marinkovic et al., 2003; Hodgson et al., 2021; Zhu et al., 2022). Therefore, the early neural RS effects identified in our work were unlikely to be associated with the processing of the linguistic (e.g., orthography, semantics, phonology) properties of visual words since these properties of unfamiliar languages were unknown to the participants. Taken together, our findings of the early neural RS effects highlight an early word-level representation of alphabetic vs. non-alphabetic languages which distinguishes words from letters/radicals but is similar for familiar or unfamiliar languages. Our results, however, do not exclude the possibility that the processing of the linguistic properties of visual words may contribute to the long-latency RS effect around 300 ms after word onset. Further processing of the linguistic properties of visual words of familiar languages may follow the early language-based categorization of visual words, though this should be tested in future research.”

**Reviewer #2 (Public review):**

Summary:

This study investigates how the human brain categorizes visual words from distinct writing systems (alphabetic vs. non-alphabetic) as a neural basis for the social-categorization function of language. Using a repetition suppression paradigm combined with electroencephalography and magnetoencephalography, the authors conducted nine experiments with independent participants to identify the neural network underlying language-based categorization, characterize its temporal dynamics, and test whether this process operates independently of linguistic properties such as semantic meaning and pronunciation.

Strengths:

(1) The study employs a well-validated design with clear control conditions and systematically manipulates key variables, including writing system, language familiarity, and native language background. The use of nine experiments with independent participant samples strengthens the reliability and replicability of the results.

(2) The work combines EEG and MEG, cross-validating findings across imaging modalities to support the reported neural effects. A combination of univariate, multivariate, and connectivity analyses is used to characterize neural responses and network interactions.

(3) Results are consistent across multiple language groups and for both familiar and unfamiliar languages, supporting the generalizability of the identified neural mechanism beyond specific languages or prior experience.

Weaknesses:

The authors provide compelling evidence that the identified neural network supports the categorization of words by language, including computations of intra-language similarity and inter-language difference. However, the conceptual framing of this finding as directly reflecting the social-categorization function of language may be premature. While the task captures spontaneous language categorization, it does not involve social evaluation or intergroup processes. The connection to social categorization is inferred from prior literature rather than demonstrated within the current experimental design. Clarifying this distinction would strengthen the conceptual precision of the manuscript.

Thank you for this important comment. In the revised Introduction and Discussion, we’ve clarified several related issues. First, prior research suggests that language can serve as a socially relevant category cue. Second, these findings imply that rapid categorization of words by language may occur in the human brain. Third, although our results identify a neural network supporting such rapid language-based categorization of visual words, they do not directly test how this process relates to social categorization of people (see Page 3-4; Page 39). Highlighting these points help delineate the scope of our findings and point to important directions for future research.

Page 3-4

“The social-categorization function of language revealed in these behavioral studies implicates that rapid categorization of words of different languages may occur in the human brain. Furthermore, the findings of infant studies (e. g., Liberman et al., 2017b) suggest that the neural process involved in categorization of words of different languages may develop even prior to the processing of linguistic properties (e.g. semantic meanings) of words. Nevertheless, up to date, there has been little neuroimaging research examining the neural mechanisms underlying automatic and fast categorization of words of different languages.”

Page 39

“Finally, it should be noted that the current work was initiated by the previous behavioral findings which suggest that language can serve as a socially relevant category cue but focused on the neural mechanisms underlying rapid language-based categorization of visual words. Although the previous findings suggest that the language-based categorization of visual words provides a cognitive basis of social categorization of people, our work did not directly test whether and how the neural processes involved in the language-based categorization of visual words are linked to social evaluation or intergroup processes which are critical for social categorization of people. To clarify this issue should promote deep comprehension of the neural mechanisms underlying the social-categorization function of language but is beyond the scope of the current study. Future research should investigate the connection between language-based categorization of words and social categorization based on other social cues (e.g., faces), which is pivotal to understanding of social interactions in real-world situations.”

**Recommendations for the authors:**

**Reviewer #2 (Recommendations for the authors):**

(1) Revise the conceptual framing to clarify the relationship between the experimental results and the proposed social-categorization function of language. If the authors wish to retain the emphasis on social categorization in the title or discussion, they should explicitly explain how the observed neural mechanisms of language-based word categorization link to social evaluation, intergroup processes, or real-world social categorization. This clarification would strengthen the conceptual coherence and justify the use of social categorization within the current study's scope.

Thank you for this and the following suggestions. In the revised Introduction and Discussion, we’ve clarified the following point: First, the findings of prior behavioral studies suggest a social-categorization function of language. Second, based on these behavioral findings, we predicted automatic and fast categorization of words by language. Our study tested this prediction using neuroimaging and investigated the neural mechanisms of language-type-based categorization of visual words. This is the main goal of our work. Third, to examine how the observed neural mechanisms of language-based word categorization link to social evaluation, intergroup processes, or real-world social categorization is important but beyond the scope of the current work. However, this is a very important question. Future research should test the connection between the neurocognitive processes involved in social categorization of people and the neural categorization of visual words by language revealed in our study. Consistently, the title of our paper “Neural categorization of visual words of alphabetic and non-alphabetic languages” and Discussion focus on contributions of our findings to understanding of the neural categorization of visual words by language rather than its connection to social categorization of people. Above all, we’ve clarified in the revision that our study was initiated by the findings of social function of language but was limited to the neural processing of visual words (see Page 3-4; Page 39). Thanks again for this comment.

Page 3-4

“The social-categorization function of language revealed in these behavioral studies implicates that rapid categorization of words of different languages may occur in the human brain. Furthermore, the findings of infant studies (e. g., Liberman et al., 2017b) suggest that the neural process involved in categorization of words of different languages may develop even prior to the processing of linguistic properties (e.g. semantic meanings) of words. Nevertheless, up to date, there has been little neuroimaging research examining the neural mechanisms underlying automatic and fast categorization of words of different languages.”

Page 39

“Finally, it should be noted that the current work was initiated by the previous behavioral findings which suggest that language can serve as a socially relevant category cue but focused on the neural mechanisms underlying rapid language-based categorization of visual words. Although the previous findings suggest that the language-based categorization of visual words provides a cognitive basis of social categorization of people, our work did not directly test whether and how the neural processes involved in the language-based categorization of visual words are linked to social evaluation or intergroup processes which are critical for social categorization of people. To clarify this issue should promote deep comprehension of the neural mechanisms underlying the social-categorization function of language but is beyond the scope of the current study. Future research should investigate the connection between language-based categorization of words and social categorization based on other social cues (e.g., faces), which is pivotal to understanding of social interactions in real-world situations.”

(2) Clarify the consistency between the reported model order (5 ms lag) and the sampling rate after downsampling (250 Hz, corresponding to 4 ms per time point). If a discrepancy exists, clearly explain how the time-series data were processed.

We clarified in the revision (see Page 53) that “because down-sampling was not applied to the GCA analyses, a 5-ms lag was used for prediction of the neural activity in one brain region using the neural activity in another brain region”.

(3) For the representational similarity analysis (RSA), report reliability measures for the representational dissimilarity matrices (e.g., split-half reliability) to verify that the observed effects are stable given the number of trials per condition.

Following this suggestion, we’ve conducted split-half reliability analyses and reported the results in the revised supplementary materials. The reliability analyses are also mentioned in the revised Discussion (see Page 40).

Page 40

“In conclusion, our EEG and MEG results revealed robust RS effects in the early neural responses to visual words of the same language. The reliability of these RS effects was confirmed across words of different familiar and unfamiliar languages, in samples of speakers with different native languages, and through split-half reliability analyses (see Supplementary Materials, Fig. S19). These effects were supported by the bilateral neural networks whose activity reflected computations of correlation distances between word pairs, capturing both intra-language similarity and inter-language differences during the categorization of visual words in alphabetic and non-alphabetic languages. Together, these findings advance our understanding of spontaneous, language-based neural categorization of visual words as a key basis of the social-categorization function of language.”

(4) Provide complete statistical information for all significant results reported in the supplementary materials, including relevant test statistics (e.g., t-values, cluster p-values) in figure legends or a supplementary results table to improve transparency.

Complete statistical information has been provided in the revised supplementary materials (see Tables S4 and S5).

(5) Streamline the presentation of the nine experiments in the main text to emphasize the core conceptual and methodological logic, potentially using a schematic overview or flowchart to improve readability.

As suggested, we’ve included an overview of the nine experiments in the revised Introduction. This overview helps understanding of the core conceptual and methodological issues in our work (see Page 6).

Page 6

“In nine experiments we recorded EEG/MEG signals from Chinese, English, and German speakers when viewing words of an alphabetic language and a non-alphabetic language (English and Chinese words, or Italian and Korean words) or of two alphabetic languages (English and German) in the Rep-Cond and Alt-Cond. We recorded EEG signals from Chinese participants to examine temporal neural dynamics of spontaneous language-based word categorization in Experiment 1. The similar paradigm was employed in Experiments 2 and 3 to investigate whether perceptual features or radical/letters of words are sufficient to generate spontaneous language-based categorization of visual words. The results in Experiment 1 were replicated in native English and German speakers in Experiments 4 and 5, respectively. Neural dynamics of categorization of words of two unlearned languages were further investigated in Chinese participants in Experiment 6. Finally, the neural networks supporting the spontaneous categorization of words of two learned or unlearned languages were localized using MEG in Chinese and English speakers in Experiments 7-9, respectively.”

(6) Strengthen the transition between the discussion of the social-categorization function of language and the neural mechanisms of visual word categorization in the introduction.

Following this suggestion, we’ve modified the Introduction to strengthen the transition between the discussion of the social-categorization function of language and research on neural mechanisms of visual word categorization (see Page 3-4).

Page 3-4

“The social-categorization function of language revealed in these behavioral studies implicates that rapid categorization of words of different languages may occur in the human brain. Furthermore, the findings of infant studies (e. g., Liberman et al., 2017b) suggest that the neural process involved in categorization of words of different languages may develop even prior to the processing of linguistic properties (e.g. semantic meanings) of words. Nevertheless, up to date, there has been little neuroimaging research examining the neural mechanisms underlying automatic and fast categorization of words of different languages.”

(7) Briefly define the repetition suppression (RS) paradigm when first mentioned (i.e., reduced neural response to repeated stimuli from the same category, reflecting categorical processing) to improve accessibility for non-specialist readers.

The RS paradigm is now defined in Introduction when being mentioned for the first time in the manuscript (see Page 5-6).

Page 5-6

“The present study investigated neural dynamics of categorization of visual words of two different (an alphabetic versus a non-alphabetic, or two different alphabetic) languages by combining EEG/MEG with a repetition suppression (RS) paradigm adopted from previous studies of social categorization of faces (Zhang et al., 2023b; Zhou et al., 2020). RS refers to the attenuation in neural responses to a repeated occurrence of stimuli that engage common neuronal populations or processes due to habituation (Grill-Spector et al., 2006). The RS paradigm consisted of an alternating condition (Alt-Cond), in which visual words of two different languages were presented alternately, and a repetition condition (Rep-Cond), in which words of one language were presented repeatedly (Fig. 1a). Neural responses to stimuli of the same category were attenuated in the Rep-Cond compared to Alt-Cond due to habituation and this RS effect disentangles the neural activities underlying categorization of faces and body silhouettes of a specific social group.”

(8) Report detailed participant demographic information, including exact age range/mean age and gender ratio for each experiment, to meet standard reporting practices in neuroscience.

We’ve modified Table S1 to include the information about exact age range/mean age and gender ratio in each experiment.

(9) Correct minor typographical and grammatical errors, including These finding (line 59) and Chinse (line 223).

These and other grammatical errors have been corrected in the revision.
